# Bringing in the citizen: Stakeholder participation in co-production processes of sustainable housing

**DOI:** 10.1007/s13280-026-02416-9

**Published:** 2026-05-07

**Authors:** Hanna Ahtosalo, Charlotta Harju, Eliisa Kylkilahti, Jani Lukkarinen, Katja Lähtinen, Henna Syrjälä

**Affiliations:** 1 University of Vaasa, Vaasa, Finland; 2 Natural Resources Institute Finland (Luke), Seinäjoki, Finland; 3 University of Helsinki, Helsinki, Finland; 4 Finnish Environment Institute (Syke), Helsinki, Finland; 5 Natural Resources Institute Finland (Luke), Helsinki, Finland

**Keywords:** Citizen participation, Co-production, Hackathon, Participatory processes, Service design, Transition arena

## Abstract

Incorporating citizen views through stakeholder engagement enhances the democratic and equitable governance of sustainability transitions. Using stakeholder theory as a conceptual frame, this study evaluates the use of different stakeholder participation processes to co-produce solutions for more sustainable housing in Finland. This study examines how citizens are positioned, levels of participation, and factors affecting success. Data were collected in three participatory processes: a service design process, a hackathon, and transition arenas, which sought to improve citizens’ opportunities for more sustainable housing. Our results show that citizens were positioned differently across the stakeholder processes. Although all processes aimed for high levels of participation—such as collaboration and empowerment—the achieved levels varied and, in some cases, remained limited. Moreover, several contextual, organizational, and process-related factors influenced the outcomes and shaped how participating stakeholders perceived citizens’ views. Addressing these challenges is crucial when selecting participatory methods for research, planning, and policy.

## Introduction

Stakeholder participation is a prerequisite for co-produce legitimate information and solutions for sustainable societies (Lang et al. 2012). Housing is a key area for sustainable development (e.g., Ogunmankinde et al. 2022), and thus, sustainability transition in housing requires consideration of environmental, social, and economic goals (Harju and Lähtinen 2022; Koskela et al. 2023). In Finland, for example, they comprise climate mitigation and biodiversity conservation, securing human health and well-being, and economic feasibility in decision-making (e.g., Lähtinen et al. 2024). Consequently, complex interactions exist among citizens, urban planners, and the built environment. Since solutions significantly affect citizens’ everyday lives, development of solutions for sustainability changes in housing requires the adoption of participatory approaches to collaborate with citizens and other stakeholders (Lyra and Lehtimäki 2013; Hamdan et al. 2021).

Development of citizen engagement methodologies adds new knowledge creation, improvements in legitimacy, and implementation of actions toward more sustainable societies (Avelino and Wittmeyer 2016; Huttunen et al. 2022). In practice, participatory co-production methods have been used to gain insights into citizen views on complex environmental issues (e.g., landscape management, climate change adaptation, and ecological restoration) from local to transnational and global scales (Fazey et al. 2018; Liu et al. 2020; Chambers et al. 2021). Here, co-production refers to any kind of citizen input in public services (Brandsen and Honigh 2018) with other stakeholders, which is motivated by deliberative democracy goals emphasizing the importance to integrate the citizen views, for example, in co-productive problem-solving (Kahane et al. 2013). As outcomes of the participatory processes, new knowledge, ideas, and evidence of the issues at hand may be gained (van Mierlo and Beers 2020).

Although citizen engagement also concerns legislative aspects, citizens as stakeholders do not have policy implementation power (Koontz 2005) beyond their immediate realm. Citizens’ concerns, experiences, and preferences may also be neglected by more powerful stakeholders (Stoll-Kleemann and Welp 2006). For example, obstacles to citizen participation in research and public processes are caused by information gaps and discrepancies, attitudes of public officials, quality of collaboration, group dynamics, representation criteria for communities, and process design (Ianniello et al. 2019). In all, previous results have shown that the benefits of citizen co-creation are undisputable, but there is a lack of information on how practical implementation of participatory processes affects their success (Leino and Puumala 2021).

Dialogue between citizens and other stakeholders (e.g., public authorities, non-governmental organizations) within well-designed processes (e.g., selection of participants, implementation, and mediation) (Kahane et al. 2013) enhances the impact and acceptability of the outcomes (e.g., Liu et al. 2020). Regarding studying the co-production of solutions for sustainability transitions in housing, Finland makes a suitable case region also from an international perspective for several reasons: First, the national carbon neutrality target for 2035 is pursued through ambitious municipal climate strategies for housing (Huovila et al. 2022; Kylkilahti et al. 2025). Second, citizens have a statutory role in municipalities participatory land-use planning processes concerning housing (Lähtinen et al. 2024). Third, Finland is among the forerunners in advancing the Sustainable Development Goals (SDGs) in Europe (D’Adamo et al. 2022).

The purpose of our research is to study participatory co-production methods in the context of decision-making for sustainable housing. The selected cases of co-production methods are a municipal service design process (e.g., Ansell and Torfing 2021; Rădulescu et al. 2025), a hackathon (e.g., Kvamsås 2021), and Transition Arenas (e.g., Loorbach and Rotmans 2010; Hölscher et al. 2019) that we have conducted in Finland. This study has threefold aims: (1) to analyze how other stakeholders position citizens in different types of participatory co-production processes, (2) to explore participatory co-production processes in terms of the levels of stakeholder participation, and (3) to identify the opportunities and challenges of the processes. In this way, our study advances understanding of how the positioning of citizens, the levels of stakeholder participation, and process-related conditions interact to shape the outcomes of participatory co-production for sustainable housing. By integrating two conceptual frameworks across three empirical cases, we provide a nuanced contribution to academia on how participatory processes can be designed so that citizens’ views are more effectively represented in sustainability transitions.

## Conceptual framework

Stakeholder theory (Freeman 1984) defines stakeholders as groups “*who influence or are being influenced by the actions of an organization.*” The initial focus of Freeman’s work is to enhance stakeholder participation in companies’ decision-making processes (e.g., Toppinen et al. 2016). However, there is strong underutilized potential for its theoretical and methodological development also in public organizations (Sarturi et al. 2023).

Integrating citizens into participatory co-production processes along with other stakeholders (e.g., companies, non-governmental organizations, public authorities) improves access to information on diverse interests and adds new knowledge for interpreting and solving the existing problems (Kahane et al. 2013). Yet, citizen participation is conceptually dynamic (Foster-Fishman et al. 2013) and may also emerge through community memberships (e.g., voluntary groups, organizations, and associations with shared interests) (Ohmer 2007; Ianniello et al. 2019). Since other-than-citizen stakeholder groups usually have more executive power (e.g., implementation of legislation, informal network relationships), combining citizen dialogues with discussions organized with other stakeholders is necessary for efficiency and impact of the co-production processes (Koontz 2005; Mansbridge et al. 2010; Kahane et al. 2013).

The success of citizen participation depends on the depth of public involvement and methodologies used in the participatory co-production processes (Rowe and Frewer 2004). In the most advanced processes, stakeholders are engaged in decision-making processes through communicative methodologies (Aakhus and Bzdak 2015). The depth of citizen participation may vary from one-way, top-down communication dominated by powerful actors to more deliberative forms of engagement, such as the co-production of win–win solutions mediated by neutral actors (e.g., researchers) (Reed et al. 2018). Accordingly, citizen and other stakeholder engagement requires sensitivity to local circumstances, genuine dialogue, and co-creative, non-hierarchical approaches in process implementation (Reed et al. 2018; Leino and Puumala 2021). Approaches include well-designed mediation by neutral actors to enhance and stabilize social interaction among participants (Scheffran 2006). Citizen participation arising from fruitful dialogues supports integrating public values into decision-making, enhancing decision quality, educating the public, building institutional trust, reducing conflicts, and enabling cost-effective choices (Beierle 1999). Moreover, perceived meaningfulness of the co-production processes adds to citizens’ motivation to engage in dialogues (Lennon et al. 2019).

To analyze the depth and success of participatory co-production processes, we adopt two conceptual frameworks: First, the Stakeholder Participation Planning Matrix (Bryson 2004; Bryson et al. 2011) to explore participatory methods with reference to levels of stakeholder participation. Second, the categorization of potential obstacles to citizen participation (Ianniello et al. 2019) to identify the opportunities and challenges of the participatory co-production processes. The Stakeholder Participation Planning Matrix (Bryson 2004; Bryson et al. 2011) is rooted in Stakeholder theory (Freeman 1984), which emphasizes the creation of public value and the consideration of stakeholders through means such as citizen engagement (Bryson et al. 2014). The matrix integrates two essential components: strategic management functions and levels of public participation. In our study, we focus on the latter. In the matrix, participation ranges from informing to empowering at five levels of, i.e., Inform, Consult, Involve, Collaborate, and Empower, of which each is associated with a specific value proposition for stakeholders. In practical utilization, the matrix enables contemplating the best engagement of different stakeholders at different stages of the process with the aims of harnessing their expertise and perspectives and also to mitigate potential conflicts to ensure smooth process progress.

**Fig. 1:**
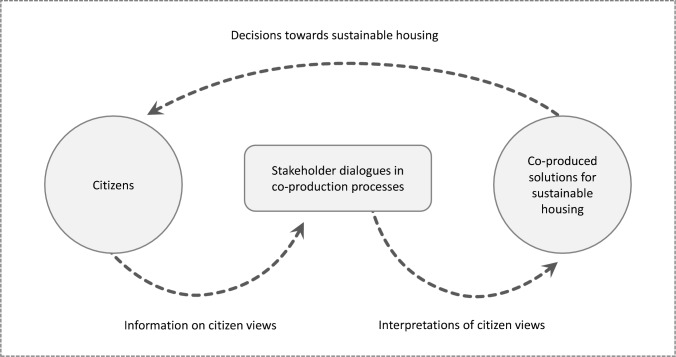
Analytical framework of the study

Figure 1 illustrates the analytical framework of the study. Information on citizen views serves as the basis for stakeholder dialogues, where these views are interpreted to co-produce solutions for sustainable housing and inform decision-making.

Furthermore, to identify the opportunities and challenges of the participatory co-production processes, we categorize potential obstacles to citizen participation (Ianniello et al. 2019). The categorization of citizen participation connects with Arnstein’s (1969) results on how citizens may be involved and gain power in public decision-making. In their systematic literature review, Ianniello et al. (2019) identified barriers to citizen participation, including contextual factors, process management patterns, and organizational arrangements. With *contextual factors*, they refer to the existing conditions influencing citizen participation, such as information gaps and discrepancies, and the attitudes of public officials. Among the *organizational arrangements* that contribute the most to successful participation are community representation criteria (e.g., participant selection) and process design (e.g., choice and implementation of tools of dialogue). *Process management patterns,* such as group dynamics and quality of collaboration, have a significant impact on the effectiveness of citizen participation. For example, group dynamics can be related to participation bias and participation of marginalized groups, while collaboration quality is related to building dialogue across diverse stakeholders. As in the case of participatory co-production processes, citizen participation emerges through interpretations made by other stakeholders or representatives of citizen communities (e.g., local activist groups, housing board members, and parishes in the transition arena process). In the following, we apply this categorization as an analytical lens to identify the challenges and opportunities within these processes.

## Materials and methods

We designed our empirical research setting around three cases involving participatory co-production processes of stakeholder participation in planning and initiating climate actions in the Finnish housing sector. These participatory co-production processes are service design (e.g., Deserti and Rizzo 2018; Ansell and Torfing 2021), a hackathon (e.g., Kvamsås 2021), and transition arenas (e.g., Loorbach and Rotmans 2010; Hölscher et al. 2019). Each of these processes positions citizen participation differently, and they were chosen with a view to opening up several avenues for co-producing sustainable housing together with multiple stakeholders—as cases they allow analytical reflection and comparison. The processes, their aims, the collected data, and the analysis methods are introduced next (see also Table 1).

**Table 1:** Data collected in the processes of municipal service design process, hackathon, and transition arenas

Process	Event	Data description	Collection time
**Municipal service design process**	Workshop 1. Orientation: Existing visions, resident participation, instruments, and technical support	120 min; Flinga whiteboard, notes All four case cities & stakeholders: 15; researchers: 7	4/9/2021 (online)
	Workshop 2. Cooperation and challenges: Citizen involvement, cooperation, networks, and related challenges	90 min; Notes All four case cities & stakeholders: 10; researchers: 8	6/17/2022 (online)
	Workshop 3. Vision 2035: Changes over time, preformed service ideas	150 min; transcription, notes, photos All four case cities & stakeholders: 7; researchers: 9	11/23/2022 (hybrid, on-site/online)
	Online questionnaire	N = 10	January 2023
	Workshop 4. Toward the service paths: Problem-solving, target groups, developers	90 min; Padlet board, transcription, notes All four case cities & stakeholders: 9; researchers: 9	2/10/2023 (online)
	Workshop 5a. City-specific service paths I: Service paths toward city-specific experiments	150 min; transcription, notes, canvases, photos Two case cities & stakeholders: 8, researchers: 8	3/22/2023 (on-site)
	Workshop 5b. City-specific service paths II: Service paths toward city-specific experiments	300 min; transcription, notes, canvases, photos One case city & stakeholders: 5; researchers: 6	5/9/2023 (on-site)
In addition, preparation materials and a 24-page living memo of researchers from the whole process			
**Hackathon**	Two-day event preparation workshop	Definition of the requirements and user group of the application to be developed: notes, photos, description of the target group (Word document), requirements specification (picture); 9 researchers	2/7–8/2023 (on-site)
	Hackathon challenge definition workshop; workshop facilitated by a partner organizer	90 min; Instructions and completed tasks on the Miro platform; 4 representatives from partner organizations and 7 researchers	2/16/2023 (online)
	Hackathon event	A 48-h innovation competition; teams’ competition ideas as PDF presentations, teams’ pitches as videos, observation data (Word), teams’ reflection posters (photos), jury evaluation forms (Excel), final feedback survey (N = 23), photographs and video recordings of the event’s activities; 12 experts, 6 jury representatives, 5 facilitators from partner organizations, 29 participants, 1 photographer, 10 researchers	9/15–17/2023 (on-site)
	Post-hackathon meeting	60 min; Agenda (Word), notes; 4 representatives from partner organizations, 6 researchers	9/29/2023 (online)
In addition, preparation materials, meeting notes, chat and e-mail messages, and memos from two researchers			
**Transition arena (suburban)**	Transition arena process of 4 workshops	21 participating stakeholders (7 from the public sector, 6 citizen activists, and 8 business representatives), 7 expert facilitators	12/1/2022–3/23/2023
	Workshop 1: Building vision and setting targets	180 min Recordings, canvases, pre-arena survey, and notes	12/1/2022 (on-site)
	Workshop 2: Backcasting transition pathways 1	180 min Recordings, notes, pathway maps (Miro + PDF)	1/12/2023 (online)
	Workshop 3: Backcasting transition pathways 2	180 min Recordings, notes, pathway maps (Miro + PDF)	2/9/2023 (online)
	Workshop 4: Lessons and actions	180 min Recordings, notes, filled canvases	3/23/2023 (on-site)
In addition, preparation materials, meeting notes, and an arena background information package			
**Transition arena (rural)**	Two-day transition arena retreat	Ten participating stakeholders (4 from the public sector, 3 citizen activists, 2 knowledge producers, 1 business representative), 5 expert facilitators	4/18–19/2023 (on-site)
	Step 1: Building vision and setting targets	90 min Recordings, canvases, pre-arena survey, notes	
	Step 2: Backcasting transition pathways	270 min (× 2) Recordings, notes, pathway maps (Miro + PDF)	
	Step 3: Lessons and actions	100 min Notes, filled canvases, pictures	
	Follow-up meeting	120 min Notes 7 participants, 3 facilitators	6/13/2023 (hybrid)
In addition, preparation materials, meeting notes, and an arena background information package			

**The service design process for municipalities aimed** to create a workshop setting to collaborate with representatives of four case municipalities (Helsinki, Joensuu, Turku, and Vantaa) which would ideally lead to co-production of solutions and a municipality toolbox, i.e., novel or improved services for sustainable housing. The invited participants from these municipalities worked with city planning, housing, and environmental issues. In practice, the workshops were methodologically similar to focus group discussion—the researchers mediated the discussions and provided a set of elicitation materials to help participants elaborate on the topic (Moisander and Valtonen 2006).

The first two online workshops focused on a generic discussion about the current state of resident participation and challenges related to citizen involvement within municipalities’ stakeholder networks (Table 1). The third workshop kicked off the actual service design process by inviting the participating stakeholders from case municipalities to define a vision collaboratively for the service design challenge. In the series of workshops, the co-produced vision was followed by service ideation and the development of these ideas into concepts for experiments. Moreover, this phase of the service design process was preceded by analyses of the case municipalities’ strategies and local challenges and citizen interviews. The workshop process, data collection, participants, and pre-analysis of municipal documents and citizen interviews are described in more detail in Kylkilahti et al.(2025).

The participatory approach involved inviting key stakeholders named by the municipality representatives or identified by the research team to participate, and using practical tools to facilitate workshop discussions, including resident personas, an applied version of the business model canvas, and a service journey map, which are derived from the co-creative service design approach (Osterwalder and Pigneur 2010; Stickdorn and Schneider 2010; Ojasalo and Ojasalo 2018; Cherry et al. 2022). Resident personas were constructed based on the analysis of the citizen interviews and diaries, and then utilized as elicitation material in the third workshop. The purpose of these personas was to concretize the various living situations and priorities of different types of residents and to help the participants to reflect on their personal experiences as residents to develop service ideas. Canvases based on service journey and business model elements were given to the participants in workshops 5a and 5b to encourage them to develop their service ideas until they were sufficiently tangible. These tools posed questions such as: *Who is the service user? Who are the relevant collaborators? Which problem does this service solve? What are the phases of the service use? In which service touchpoints is the service user encountered?*

**The aim of the hackathon** was to employ a collaborative design method to generate ideas and early-stage prototypes for a gamified application to promote sustainable housing. Hackathons are intensive, time-constrained innovation events where small, interdisciplinary teams collaboratively design and prototype solutions typically ranging from one to three days (Komssi et al. 2015; Trainer et al. 2016; Richterich 2019). Beyond serving as means for rapid software development, hackathons also function as sociotechnical arenas within broader organizational and innovation ecosystems, fostering creativity, learning, and collaboration where the goal may be to ideate or conceptualize potential solutions rather than to produce fully functional prototypes (Nolte et al. 2018; Perng et al. 2018; Flus and Hurst 2021).

The hackathon process involved multiple phases. Seven months prior to the hackathon, in a two-day workshop, researchers analyzed data and identified detached house owners as the target group. This session also established functional requirements, categorizing them into three priority levels: must-have, may-have, and nice-to-have. To ensure a multidisciplinary participant pool, the research team, together with the hackathon facilitation company, strategized recruitment efforts and defined the desired expertise for the event. This formulated a concise challenge statement to guide participants throughout the hackathon weekend.

The hackathon event was a 48-h innovation competition held in Helsinki in September 2023. It followed the typical hackathon structure of ideation, team formation, and prototype development (Komssi et al. 2015; Gama 2017; Richterich 2019). However, this event focused on refining multidisciplinary ideas into concrete and illustrative concepts (see also, Irani 2015; Richterich 2019; Flus and Hurst 2021). The prioritization of persuasive presentations and conceptual viability over functional code is characteristic of events focusing design, learning, and entrepreneurial outcomes, often involving diverse skill sets such as designers, artists, and business experts (Nolte et al. 2018; Richterich 2019; Flus and Hurst 2021). The event brought together 29 participants and was supported by a multidisciplinary team consisting of 12 subject matter experts (e.g., heat pumps and maintenance, energy retrofits, heating solutions, building physics, consumer behavior, and gamification), five facilitators from partner organizations, ten researchers facilitating the process (some of whom also contributed as experts), and a photographer.

Over the weekend, seven application concepts were developed. These were evaluated by a six-member jury composed of three experts in detached housing (NGOs), an expert representing the research team, one specialist in application development and gamification, and an assisting researcher without voting rights. The jury selected the three best concepts—the winning teams received monetary awards, and the most promising concept was offered support to develop a publicly available version.

**The transition arena processes aimed** at tackling the challenges of climate action in the housing sector. The arenas were designed as facilitated co-production workshop series, with stakeholders of various backgrounds (public sector, business, citizen activists). Transition arenas provide a systemic framework for stakeholder deliberation accelerating action on a defined sustainability issue (Loorbach 2010; Hölscher et al. 2019). The core idea behind the transition arenas is to combine participatory co-design processes with the future studies methods to produce actionable “transition pathways,” deliver contextually rooted recommendations, and encourage experimentation (Frantzeskaki et al. 2018). While transition arenas are promoted as providing actionable and policy-relevant insights (Hyysalo et al. 2019) and participatory learning (Lähteenoja 2022), they are also criticized for tendencies toward “elitist capture” and a “post-political approach” because of reliance on expert knowledge and lack of direct, democratic participation of citizens (Voß et al. 2009; de Geus et al. 2022; Lukkarinen et al. 2025). Both arenas proceeded in three main phases: defining of transition challenges and goals, developing transition pathways, and prioritizing the most important actions. Finally, recommendations were publicized to generate interest on the issue (see Lukkarinen et al. 2023a, b). Both processes have been presented in Lukkarinen et al. (2025).

A different approach was taken to document each of the three participatory co-production processes to serve their purposes (Table 1). All the data generation followed an ethnographic approach (Moisander and Valtonen 2006). In addition to formal data, the analysis also drew on researchers’ field notes, reflections, and informal discussions gathered throughout the processes. Table 1 presents the collected data in each process.

Each participatory case process was analyzed separately by researchers who had been involved in the processes. For the purpose of this study, the results were combined to respond to the research questions of the study. The data were analyzed with content analysis, and as theoretical tools, we adopted the Stakeholder Participation Planning Matrix (Bryson 2004; Bryson et al. 2011) to explore the levels of stakeholder participation, and categorization of potential obstacles for citizen participation (Ianniello et al. 2019) to identify the opportunities and challenges of the participatory processes.

## Results

### Positioning of citizens and levels of stakeholder participation in different processes

Each of the three studied participatory co-production processes aimed to create knowledge and solutions to enhance citizen participation for sustainable transitions in housing. However, instead of posing questions directly to individual citizens, an indirect approach to citizen participation was employed through the involvement of different stakeholders in the co-production processes. Consequently, in each process, citizens were positioned differently. In line with the analytical framework (Fig. 1), we next examine what kind of information on citizen views was provided, how citizens were understood, and how their views were interpreted by different stakeholders when referring to citizen-related discussions. In addition, we identify the intended and achieved levels of stakeholder participation (Bryson 2004) in the three case processes.

#### Municipal service design process

The main issue tackled in the workshops was identifying ways to integrate climate action into municipal activities in the context of housing and construction. Thus, citizens were primarily positioned as residents or occupants of buildings—they were seen as the ultimate service users. In practice, this resulted in discussing services such as counseling and permitting services for new construction and renovation projects that are managed and operated only by certain units within municipalities. Indeed, according to our analysis, citizens were described as residents who are seldom in direct relationships with the municipality. The municipal representatives struggled to recognize touchpoints and possibilities to directly interact with residents. Instead, their views are interpreted and transmitted via other stakeholders such as companies, public decision-makers, or building owners. Throughout the process, a shared understanding of the need to increase the ease of sustainable housing solutions for residents was highlighted. Moreover, it was identified that while municipalities have complex organizational structures and decision-making processes that are difficult for residents to understand, it is challenging to motivate them to participate in, for example, commenting on city plans. Thus, the service design process, together with NGOs and researchers, focused on developing services that would support the activity level of residents and new ways of interacting.

The intended level of stakeholder participation (Bryson 2004) in the municipality service design process was to *collaborate* with municipality actors and other stakeholders identified within the process. This target was partly met; however, the achieved collaboration did not occur to the intended extent, since not all of the recognized key stakeholders engaged in the process. In addition, the process was designed to *empower* the municipalities to take action and bear responsibility in the development of the services after the workshop phase. However, the shift to empowerment was not immediately achieved. This might be due to changes in operational environments, especially the stagnant construction business. Also, it seemed that within the public sector it is not easy to align resources with the aims of the organization.

#### Hackathon

In the hackathon, a citizen was understood to be a resident who makes energy-related everyday housing decisions. A key challenge was providing reliable and accessible information on energy and climate issues in a format that appeals to citizens. The goal was to make the environmental and financial benefits of sustainable housing more tangible, thereby encouraging residents to adopt more climate-conscious lifestyles. To address this challenge, participants aimed to develop gamified solutions to help homeowners make climate-wise decisions about home maintenance and energy renovations.

From the perspective of stakeholder participation, none of the hackathon participants were homeowners living in detached houses, which was the target group for the proposed solution. Therefore, during the ideation and design process, researchers provided a summary of the results from a representative citizen survey (see survey information in Ruokamo 2024a; b) including demographic and contextual insights, as well as information on climate attitudes, motivations for energy renovations, and daily practices among homeowners of detached houses in Finland. The co-production solutions were specifically designed for a Finnish “average detached house dweller,” based on expert input. Also, to support the design work, background information was also provided on the detached homeowners’ everyday choices, their perspectives on climate-wise living, and specific information regarding emissions originating from detached houses in Finland.

In conclusion, the hackathon participants interpreted citizen participation based on the information they received from researchers, while the suitability of the proposed ideas for the target group was ultimately assessed by a jury. The intended level of stakeholder participation was to *collaborate* with NGO stakeholders and to involve them in the innovation process, emphasizing that the hackathon was not intended as an one-off event, but rather as a starting point for ongoing development. Though *empowerment* was also a desired outcome, it will only be realized when the proposed ideas are translated into concrete actions. In the case of the hackathon, this includes an application maintained and developed further by a specific stakeholder organization. To facilitate this transition, researchers act in a mediating role to develop a first prototype of the selected idea, in collaboration with the winning hackathon team, an application development team, and an expert panel of relevant stakeholders.

#### Transition arenas

The transition arenas viewed citizen participation under the umbrella term “energy literacy.” Energy literacy refers to the capacity of different citizen groups to understand energy measurements and metrics and correctly use devices and equipment that contribute to energy consumption at a household level (Van den Broek 2019). However, while the lessons consistently revolved around providing information support at the household level, energy literacy was approached in contradictory terms depending on whether it was understood as emancipation or victimization of citizen groups. Another concrete lesson on citizen participation was the identified need for practical peer-to-peer support approached by identifying local champions: housing units engaged in innovative energy renovations proactive energy communities.

The objective of the transition arenas was to *collaborate* with stakeholders that influence on policy and planning developments to change the institutional conditions of citizen participation. Collaboration was partially successful, as the lessons were taken forward at the implementation level of energy counseling and advice, while collaboration with more strategic municipal climate governance proved difficult.

Overall, the three processes positioned citizens quite differently, focusing on different areas of citizens in climate action (Table 2).

**Table 2:** Role of a mediated citizen in the three processes

	Municipal service design process	Hackathon	Transition arenas
Understanding of what a “citizen” is	Citizen as a subject of municipal planning	Citizen as a decision-maker at a household level	Citizen as a member of a community
Involvement of citizens	Not directly present in the process, understood via stakeholder experiences, NGO representatives and collected data	Not directly present in the process, understood via collected data, invited as solution testers at later stages of the development process	Hand-picked experts, including representatives of community groups and NGOs
Identified solutions	Improved municipal service concepts to engage citizens in planning	Gamified and actionable digital application on housing decisions	Identified barriers and structural challenges for citizen agency

### Opportunities and challenges in the processes

In accordance with Ianniello et al. (2019), we categorized the opportunities and challenges of the participatory co-production processes into contextual factors, organizational arrangements, and process management patterns, as described in the following.

#### Municipal service design process

*Contextual factors* refer to the conditions influencing citizen participation (Ianniello 2019). In the municipal service design process, they were related to issues arising from the changing operational environment, such as the stagnant construction industry and changes in the political atmosphere. Furthermore, some participants in the process expressed critical attitudes about the municipality’s possibilities to act, which influenced the conditions for positioning citizens as stakeholders with an active decision-maker role.

*Challenges and opportunities in developing climate-wise services related to organizational arrangements highlight organizational decisions, the organizational adaptation process, and organizational consequences *(Ianniello 2019)*. In the municipal service design process, these were particularly strongly present. We identified several organizational arrangements, including municipalities’ changing targets and focus areas, types and sizes of municipalities, and different timeframes of action between municipalities, residents, and funding agencies. For example, a typical timeframe issue is that residents and housing companies operate mainly in the short term, requiring immediate actions from municipalities, while city planning is long term oriented; for example, energy zoning plans are done over the course of twenty years. Moreover, the organizational arrangements were related to the ways of developing methods for stakeholder participation in municipalities, limited power of municipalities, structures for company cooperation in municipalities, and pre-existing national, regional, and municipal services. For example, possibilities to support active residents were considered challenging.*

The most relevant factors related to *process management* included high variation among the participants and the non-participation of key stakeholder representatives in the dialogues. These challenged the process by causing discontinuities in the information flow and design process. Moreover, the process was interrupted by participants who questioned the co-produced tasks at hand, and also to some extent by the fact that some participants changed their own views during the process. However, these factors also allowed new opportunities to emerge and encouraged participants to justify their approaches in more detail, which may have pushed them to establish common ground more clearly.

#### Hackathon

In the hackathon, the *contextual factors* influencing the positioning of citizens as stakeholders included the extent, availability, and scope of background materials and time restrictions. To introduce participants to sustainable housing, research findings, and application requirements, researchers prepared a comprehensive 32-slide presentation. While this provided a solid foundation, the amount of new information may have been overwhelming for participants unfamiliar with the topic, making it difficult to fully grasp all aspects within the limited timeframe. Due to time constraints, not all teams could consult every expert, and some participants felt that they could have contributed better if they had had more time. Defining the challenge scope helped guide the teams but it also constrained innovation, as most solutions were designed in accordance with the researchers’ initial requirements.

The *organizational arrangements* included process design aspects regarding the facilitation partner, participant selection, and legal and regulatory factors. The hackathon method and the facilitation partner company were chosen years before the event. Once the collaboration began, challenges emerged in defining responsibilities between the organizing partners, researchers, and an outsourced hackathon facilitation company. Communication gaps led to unclear decision-making, delays in task execution, and uncertainty regarding event ownership. The selection process required participants to submit an online application explaining their interest in the event and suitability. Although the recruitment process was open to Finnish and English speakers, most promotional materials were in English. This likely increased the number of non-Finnish-speaking participants, which had implications for group dynamics and the development of locally relevant solutions. Legal and regulatory factors also played a role. While agreements ensured fair treatment of participants and granted intellectual property rights to the organizer, the contractual process introduced bureaucracy that may have discouraged some participants. Some attendees expressed concerns about the speed of the agreement-signing process and transparency regarding ownership of ideas.

One major factor related to *process management* issues, such as group dynamics and quality of collaboration, was the composition of the participant group. The invitation was open to a broad audience, including students, professionals, startups, and hobbyists. However, recruitment efforts primarily targeted student communities, which is reflected in the final composition of participants. While the selection process aimed to ensure a diverse group with relevant skills, including expertise in application development, user-centered design, understanding of human behavior, and Finnish language, most participants were relatively young, many were exchange students, and very few had direct experience living in Finnish detached houses, an aspect relevant to the hackathon’s theme. The idea generation and planning processes could have been enhanced by including a human behavior expert in each team, similar to individuals with first-hand experience of living in a Finnish detached house.

Team formation was another critical factor. Teams were assembled based on applicants’ profiles, often grouping individuals who had never met before. This sometimes led to mismatches in skill levels and team interests, affecting collaboration quality. Despite these challenges, team-building efforts were largely successful. Facilitators implemented various strategies to foster group cohesion, and by the end of the event, motivated and functional teams had formed, working collaboratively on their assigned challenge.

#### Transition arenas

In the transition arena processes, the *contextual factors* were at the core of process development. Facilitators combined material packages identifying the most important aspects impacting sustainable housing in the suburban and rural contexts. In addition, attempts were made to integrate the policy co-production processes into the current development activities of the regions. However, as the processes developed, officials became reluctant to engage in transition arena interventions, which potentially reflected a governance mismatch. In both contexts, climate work was considered as monitoring and guiding municipal-level developments, while the transition arenas were tied to more specific energy planning challenges. Both cases showed the importance of officials who are interested in engaging in co-production activities. A special unit focused on counseling and information generation in Helsinki as well as a project team developing energy action under the regional council in Joensuu took a strong ownership in the processes, helping to bridge the co-creative lessons into official policy developments. Overall, the processes were designed to engage different viewpoints on the complex issue of energy planning, aiming to support the learning of stakeholders who had little previous information on this issue.

*Organizational arrangements* provided opportunities for solution-oriented dialogues in both processes, while relying on the idea of invitation-based participation. The stakeholders were selected to form broad groups of actors familiar with different insights into planning, knowledge generation, practical action, and citizen engagement to consider the challenges and opportunities of sustainable housing. Therefore, the representation requirements were not demographic but rather based on diversity of viewpoints. However, such representation also created challenges, as not all the stakeholders had a direct connection to the community in question, and limiting participation to 20 stakeholders narrowed the scope of co-production.

The process design of the transition arenas was highly structured. Both processes started with the identification of the main contextual challenges and opportunities for sustainable housing, continued by setting a set of transition targets and co-producing pathways for desirable actions, and closed by identifying key recommendations. However, the mediation of interaction was resource intensive, as the structured implementation required detailed planning. Furthermore, two of the four workshop sessions in Helsinki took place online, which complicated interaction by eliminating opportunities for spontaneous exchanges. Nevertheless, the organizers ensured that there were no technological boundaries for participation.

Finally, *process management* in the transition arenas relied on multiple facilitation dynamics that included rounds of small group discussions, large group exchanges, individual work, and prioritization. Mediators paid careful attention to balancing the speech time of individual stakeholders and preventing participants from dominating the process. However, the emphasis on solutions did not resonate with all participating stakeholders. Overall, the quality of stakeholder collaboration was improved by not requiring a full consensus on the proposed actions, but rather valuing the convergence of diverse viewpoints. Convergence was considered especially valuable, as the issue of justice remains under-conceptualized in these sustainable housing contexts.

## Discussion and conclusions

The study focused on three participatory co-production processes in decision-making for sustainable housing in Finland. We approached citizen participation by applying the Stakeholder Participation Planning Matrix (Bryson 2004; Bryson et al. 2011) and the categorization of potential obstacles to citizen participation (Ianniello et al. 2019). This combination provides a basis for discussing how co-production processes can support inclusive and informed decision-making.

First, the research provided three distinct framings of citizen participation, which shape how citizens’ perspectives on sustainable housing are interpreted: as residents for whom climate-wise housing should be enabled (municipal service design), as household members who continuously make conscious or unconscious decisions (hackathon), and as members of local communities that are targets of policies. The different conceptualizations show that citizen positioning is strongly influenced by the context and objectives of co-production processes. Importantly, the way citizens are positioned shapes expectations regarding their role in sustainability transitions. For example, in the hackathon, responsibility for implementing solutions was implicitly shifted toward citizens themselves, while in the transition arenas, citizens were framed as targets of public support. These differences demonstrate how stakeholder interpretations of citizens not only influence how citizen views are represented in co-production processes but also the distribution of responsibility and required support among stakeholders in the policy.

Second, the research explored the intended and achieved levels of engagement of the participatory methods with reference to the levels of stakeholder participation (Bryson 2004; Bryson et al. 2011). All processes aimed at high levels of stakeholder participation to enable collaboration and empowerment. However, the achieved levels of engagement varied, as the implementation of lessons and outputs requires time, resources, and involvement of additional stakeholders. Our results support earlier research findings: Success in stakeholder engagement is dependent on, for example, fit between goals and methods, selection of the stakeholders, and the motivation and power of actors to put co-created solutions into action (e.g., Scheffran 2006; Reed et al. 2018; Lennon et al. 2019).

Third, the research identified opportunities and challenges of the processes. The opportunities and challenges were categorized into contextual factors, organizational arrangements, and process management patterns (Ianniello et al. 2019), which also served as a tool to understand how different factors influence the conditions for successful participation among stakeholders.

*Contextual factors* created both opportunities and challenges in the processes. The opportunities consisted of well-defined targets for co-production and co-learning by linking diverse backgrounds, while the challenges included time constraints and linking process outputs to policy continuums. Meanwhile, extensive background information packages, attitudes of participating stakeholders, and changes in regulation created both opportunities and challenges for participation.

Several aspects related to *organizational arrangements* impacted the implementation of the processes, including different characteristics of the participating municipalities, collaboration with a facilitation company and recruitment of participants in the hackathon, as well as the limited number of participants narrowed direct citizen perspectives in the transition arena process.

Challenges regarding *process management* were mainly about group dynamics. The high variation among participants in each process generated new and enriching ideas but also led to discontinuities. Overall, collaboration quality, emerging from collaborative interactions among the actors participating in co-production processes (Bremer and Meisch 2017), was a critical factor in our processes. From the perspective of stakeholder theory, this relates to the challenge of finding a balance between stakeholder representation, societal power relationships, and opportunities to elicit the views of diverse stakeholders through dialogue mediation and communication (e.g., Kahane et al. 2013; Sarturi et al. 2023).

All cases aimed at co-producing solutions, which could be later adopted and further developed by stakeholder organizations and eventually benefit citizens. While valuable ideas were created, the question of who would take responsibility for the follow-up and further development of the solutions remained unaddressed. Hence, another key challenge is how to achieve concrete outcomes and continue working on the solutions after the co-production processes (e.g., Koontz 2005).

Methodologically, considering participatory co-production processes as mode of data collection brings challenges of participation bias, as individuals from already active stakeholder groups become over-represented. On the one hand, there is a need to balance between professional stakeholders and citizens with more diverse backgrounds and values (e.g., Kahane et al. 2013). On the other hand, there is a challenge of how well selected participants represent or are capable of making interpretations of the views of citizens, and especially those of minorities or vulnerable groups (e.g., Mansbridge et al. 2010). Earlier research has shown that in participatory co-production processes the views of citizens may be neglected by the stakeholders with more decision-making power (Stoll-Kleemann and Welp 2006). The cases provide different insights in managing the biases by providing adequate background materials, mediating the dialogue between the groups, and verifying results.

Overall, the analysis shows that focusing on contextual factors, organizational arrangements and process management (Ianniello et al. 2019) is central in enabling more in-depth participation (Bryson 2004; Bryson et al. 2011). However, it is important to use these approaches reflexively and in relation to the context, already *ex ante*, as the diverse challenges are highly situational. The methods employed must fit the issue to be solved. As public engagement is a prerequisite for sustainability transitions and essential for diversifying knowledge and enhancing legitimacy (Lang et al. 2012; Lyra and Lehtimäki 2013; Hamdan et al. 2021; Huttunen et al. 2022), understanding and overcoming these challenges are crucial when selecting participatory methods for future research, planning, and policy development to ensure socially acceptable solutions in sustainable housing.

The limitations of this study are related to the differences between the three participatory co-production process cases (e.g., varying goals, participating stakeholders, different timeframes), which hinder possibilities to compare them effectively. Furthermore, as the context of this study is Finland, this might limit the generalizability of the results to other countries with different political or cultural contexts. However, these limitations provide avenues for future research. For example, future studies could compare participatory co-production processes across different national or regional contexts to explore how institutional, cultural, and political factors influence participatory methods and their outcomes. Additionally, future research could investigate the methodological development of stakeholder participation to ensure better representation of marginalized or underrepresented citizen groups in participatory co-production processes and as decision-makers in sustainability transition.

## Data Availability

The data are confidential and therefore not publicly available.
